# CLDN-2 Expression Aligns with Invasion-Associated Epithelial Remodeling in Colorectal Cancer

**DOI:** 10.3390/cancers18111772

**Published:** 2026-05-28

**Authors:** Adam R. Markowski, Anna J. Sadowska, Konstancja Mantiuk, Wiktoria Romańczyk, Anna Pryczynicz, Katarzyna Guzińska-Ustymowicz

**Affiliations:** 1 Department of Hypertensiology, Gastroenterology and Internal Medicine, Medical University of Bialystok, 14 Żurawia Street, 15-540 Bialystok, Poland; 2 Department of Cardiology and Internal Medicine, Provincial Welded Hospital in Bialystok, 26 Maria Skłodowska-Curie Street, 15-950 Bialystok, Poland; anka.m@onet.pl; 3 Medical University of Warsaw, 61 Żwirki i Wigury Street, 02-091 Warsaw, Poland; s082766@student.wum.edu.pl; 4 Department of General Pathomorphology, Medical University of Bialystok, 13 Waszyngtona Street, 15-269 Bialystok, Poland; 33702@student.umb.edu.pl (W.R.); anna.pryczynicz@umb.edu.pl (A.P.); kguzinska74@gmail.com (K.G.-U.)

**Keywords:** Claudin-2, colorectal cancer, tumor budding, E-cadherin, epithelial adhesion remodeling, nodal dissemination, lymphovascular invasion, Immunoscore

## Abstract

Background: Remodeling of epithelial junctional architecture contributes to colorectal cancer (CRC) progression; however, the spatial organization linking tight-junction components to early dissemination remains incompletely characterized. Claudin-2 (CLDN-2) is frequently upregulated in CRC, yet whether it is associated with compartment-specific epithelial remodeling has not been systematically examined. Methods: In a retrospective single-center cohort of 54 surgically resected CRCs, we integrated clinicopathological variables, quantitative tumor budding counts, compartment-specific membranous E-cadherin expression, lymphovascular invasion, lymphoid follicles, and immune-cell densities. Analyses focused on spatial structural relationships within the tumor. Results: Higher CLDN-2 expression was enriched among node-positive tumors and advanced TNM stages. CLDN-2–higher tumors exhibited increased tumor budding and spatially selective adhesion remodeling, characterized by reduced membranous E–cadherin at the invasive front and budding sites, with more preserved membranous epithelial organization within metastatic lymph-node deposits. Descriptive co-occurrence and correlation analyses demonstrated concordant spatial relationships among CLDN-2 expression, tumor budding, nodal involvement, lymphovascular invasion, and compartment-specific E-cadherin patterns. In contrast, immune-related parameters showed weaker differentiation across CLDN-2 strata. Conclusions: CLDN-2 expression is associated with spatial epithelial remodeling in colorectal cancer, characterized by compartment-specific adhesion changes and increased microinvasive activity. The findings support a model in which CLDN-2 expression aligns with an invasion-associated epithelial configuration linked to tumor budding and nodal dissemination. These observations warrant validation in independent cohorts with outcome data.

## Simple Summary

Colorectal cancer progression involves changes in how tumor cells are organized and interact with each other. However, the structural features that accompany early spread are not fully understood. In this study, we examined the expression of a protein called Claudin-2 in tumor samples and analyzed its relationship with features of early dissemination. We found that higher Claudin-2 expression is associated with patterns linked to early spread, including tumor budding and spatial changes in cell adhesion across different tumor regions. These patterns showed limited alignment with measures of immune-cell infiltration, suggesting that epithelial organization and immune contexture may represent partially independent aspects of tumor biology. Overall, our findings suggest that Claudin-2 is associated with a specific structural organization of the tumor that may accompany early dissemination. These observations may help guide further biological and translational studies.

## 1. Introduction

Colorectal cancer (CRC) progression from localized neoplasia to invasive and metastatic disease is driven not only by oncogenic alterations but also by coordinated remodeling of epithelial architecture. The transition to tissue invasion and nodal dissemination requires structural reorganization of junctional complexes that regulate intercellular cohesion, polarity, and paracellular permeability [1,2]. Under physiological conditions, tight junctions and adherens junctions undergo dynamic turnover while preserving barrier integrity [3,4]. In cancer, however, similar plasticity may enable localized loosening of epithelial cohesion and cellular detachment without necessarily inducing global dedifferentiation [5,6]. Spatial analyses in CRC support this context-dependent model, demonstrating that loss of cohesion is often restricted to invasive niches (particularly tumor budding areas) whereas epithelial characteristics may remain spatially heterogeneous across different tumor-associated compartments [7]. Defining the architectural programs that enable this selective and compartment-specific remodeling remains a central challenge in CRC biology.

Claudins constitute the structural backbone of tight junction strands and can maintain barrier function even in the absence of other junctional components [8]. Claudin-2 (CLDN-2), one of the earliest characterized claudins, is physiologically expressed in normal colonic epithelium, predominantly in undifferentiated crypt cells [9,10,11]. Multiple studies have demonstrated CLDN-2 overexpression in CRC at both mRNA and protein levels [9,10,11,12,13,14,15,16,17,18,19,20], and its presence in colorectal adenomas suggests involvement early in neoplastic development [10,21,22]. Functionally, increased CLDN-2 expression has been linked to altered barrier properties, inflammatory signaling, reactive oxygen species production, and activation of proliferative pathways [9]. However, the interpretation of CLDN-2 expression in CRC remains unresolved. Reported associations with advanced stage and inflammatory contexts suggest that CLDN-2 may reflect broader tumor microenvironmental or progression-related states rather than a distinct structural axis [23,24]. In addition, preserved epithelial traits at metastatic sites are often interpreted within epithelial–mesenchymal transition (EMT) and mesenchymal–epithelial transition (MET) frameworks without spatial resolution, potentially oversimplifying the dynamic and compartment-specific nature of adhesion remodeling [7,25].

In parallel, the tumor immune microenvironment represents an additional dimension of CRC organization beyond conventional TNM staging. Quantitative immune profiling, exemplified by the Immunoscore, captures cytotoxic T-cell density at the tumor center and invasive front and has demonstrated prognostic value in large CRC cohorts [26,27,28]. However, whether epithelial barrier remodeling programs intersect with immune-based stratification frameworks (or instead define partially independent axes of tumor organization) remains unclear [29,30]. In particular, the relationships among CLDN-2 expression, spatial modulation of membranous E-cadherin, tumor budding activity, lymphovascular invasion, immune-cell infiltration, and early nodal dissemination have not been systematically examined in human CRC.

The aim of this study was to determine whether CLDN-2 expression is associated with invasion-related spatial epithelial remodeling in colorectal cancer, characterized by compartment-specific remodeling of membranous E-cadherin, increased tumor budding, and alignment with early nodal dissemination. By focusing on spatial relationships within the tumor, this study seeks to clarify how epithelial organization relates to early dissemination.

## 2. Materials and Methods

### 2.1. Patients

This retrospective study included 54 consecutive patients undergoing elective colorectal resection for primary colorectal cancer (CRC) at a single tertiary center. Patients with synchronous malignancies, prior neoadjuvant therapy, or incomplete clinicopathological data were excluded. CRC diagnosis was established by colonoscopy with histopathological confirmation and radiological staging.

### 2.2. Histopathology

Formalin-fixed, paraffin-embedded (FFPE) tumor specimens were evaluated on hematoxylin and eosin (H and E)-stained sections. Tumors were staged according to the 8th edition of the AJCC TNM classification [31]. Tumor location was recorded as previously described [32].

Tumor budding was assessed according to International Tumor Budding Consensus Conference (ITBCC) guidelines in a single hotspot (0.785 mm^2^ at 20× magnification) [33]. The number of tumor buds (NoB) was quantified. For analyses requiring dichotomization, high tumor budding was defined as >2 buds within the hotspot. Complementary invasion-related parameters—tumor budding foci (TBF), poorly differentiated clusters (PDC), and poorly differentiated components (POR)—were recorded using established semi-quantitative criteria [33].

Lymphoid follicles were identified as ectopic lymphoid aggregates located within or adjacent to tumor tissue and recorded as present or absent [33].

### 2.3. Immunohistochemistry

E-cadherin and Claudin-2 (CLDN-2) expression were assessed on FFPE sections using standard immunohistochemical protocols.

E-cadherin staining was performed using a standardized avidin–biotin complex method. Membranous expression was assessed semi-quantitatively (0–3) in predefined tumor compartments, including the tumor center, invasive front, tumor budding areas, and metastatic deposits within regional lymph nodes in cases with histologically confirmed nodal metastases, as previously described [7]. Lower scores (0–1) were interpreted as reduced membranous expression, whereas higher scores (2–3) reflected preserved membranous localization. All slides were evaluated independently by two pathologists blinded to clinicopathological data, with discordant cases resolved by consensus.

Claudin-2 immunostaining was performed on 5 μm FFPE sections following deparaffinization and citrate-based antigen retrieval (10 mM, pH 6.0). Sections were incubated with rabbit monoclonal anti-Claudin-2 antibody (clone E1H9O, 1:100; Cell Signaling Technology, Danvers, MA, USA; catalog no. 13255). Membranous staining intensity was scored on a four-tier scale (0–3). Protein expression was evaluated in ten randomly selected high-power fields by two blinded observers, with discrepancies resolved by consensus. For comparative analyses, CLDN-2 expression was categorized as lower (score 2) and higher (score 3), as no tumors with absent expression were identified.

### 2.4. Statistical Analysis

Statistical analyses were performed using Statistica version 14.1 (TIBCO Software Inc., Palo Alto, CA, USA) and supplementary procedures implemented in Python 3.11 (scikit-learn 1.3.2). Continuous variables are presented as median (interquartile range, IQR). Between-group comparisons were performed using the Mann–Whitney U test for continuous variables and the chi-square or Fisher’s exact test for categorical variables. Correlations were assessed using Spearman’s ρ.

**Figure 1:**
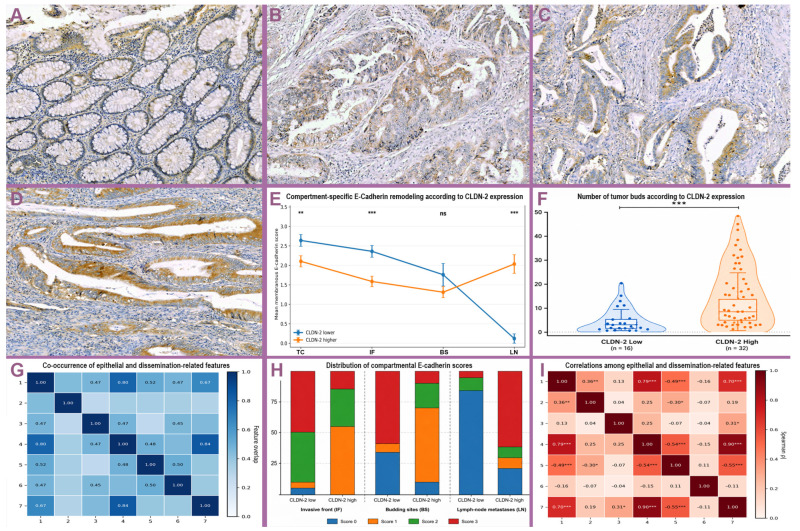
Spatial epithelial remodeling associated with CLDN-2 expression in colorectal cancer. (**A**–**D**) Representative immunohistochemical patterns of CLDN-2 expression across normal colonic epithelium and colorectal carcinoma compartments, illustrating progressive epithelial disorganization and heterogeneous membranous/cytoplasmic staining distribution within invasive tumor regions. (**E**) Compartment-specific membranous E-cadherin expression according to CLDN-2 status across tumor center (TC), invasive front (IF), tumor budding sites (BS), and metastatic lymph-node deposits (LN). CLDN-2–higher tumors demonstrate reduced membranous E-cadherin expression at invasion-related compartments, whereas metastatic lymph-node deposits retain more preserved membranous epithelial organization. (**F**) Number of tumor buds according to CLDN-2 expression status. CLDN-2–higher tumors show increased tumor budding burden and broader distribution of budding counts. (**G**) Co-occurrence of epithelial and dissemination-related features. Matrix illustrates overlap among invasion-associated epithelial variables and dissemination-related clinicopathological features. Variables: (1) higher CLDN-2 expression; (2) NoB > 2; (3) lymphovascular invasion (LVI+); (4) nodal involvement (N+); (5) low membranous E-cadherin expression at the invasive front (E-CDH-IF low); (6) low membranous E-cadherin expression in budding sites (E-CDH-BS low); (7) preserved membranous E-cadherin expression in regional lymph-node compartments (E-CDH-LN high). (**H**) Distribution of compartmental E-cadherin scores according to CLDN-2 expression status across invasive front, tumor budding sites, and metastatic lymph-node deposits. Progressive redistribution toward lower membranous E-cadherin scores is observed within invasion-related compartments of CLDN-2–higher tumors, whereas metastatic lymph-node deposits retain membranous E-cadherin expression more frequently than invasion-related tumor regions. (**I**) Correlations among epithelial and dissemination-related features. Correlation matrix demonstrating relationships between epithelial remodeling variables, tumor budding, lymphovascular invasion, nodal involvement, and CLDN-2 expression. Variables are coded as (1) CLDN-2; (2) NoB; (3) LVI; (4) N stage; (5) E-CDH-IF; (6) E-CDH-BS; (7) E-CDH-LN. Correlation magnitudes should be interpreted in view of the modest cohort size. Statistical significance is indicated as follows: * *p* < 0.05; ** *p* < 0.01; *** *p* < 0.001; ns, not significant.

Pairwise associations among epithelial, invasion-related, and immune variables were evaluated using descriptive comparisons and Spearman correlation analyses. For Figure 1G, binary epithelial and dissemination-related features were summarized using a co-occurrence matrix based on feature overlap. For Figure 1I, pairwise Spearman correlations were calculated among selected epithelial and invasion-related variables.

Given the modest cohort size and exploratory single-center design, the analytical approach was intended to characterize spatial structural relationships rather than to establish independent predictive or causal effects. Accordingly, all statistical comparisons, co-occurrence analyses, and correlation structures should be interpreted as internally descriptive and hypothesis-generating. No external validation, formal adjustment for confounding, or predictive modeling was performed. Analyses involving E-CDH-LN variables should be interpreted cautiously because lymph-node E-cadherin assessment was available only in cases with histologically confirmed nodal metastases. Nominal *p*-values should therefore be interpreted in conjunction with effect-size consistency, spatial coherence, and biological plausibility.

## 3. Results

### 3.1. Cohort Characteristics

The study cohort comprised 54 patients with primary colorectal cancer (CRC), with balanced sex distribution and a predominance of T3 tumors (94.45%) (Table 1). Lymph-node metastases were present in 46.3% of cases, and most tumors were classified as TNM stage II–III. Histopathological assessment demonstrated frequent lymphovascular invasion (62.96%), whereas perineural invasion was uncommon (7.41%). Tumor budding activity was generally low to moderate (median number of buds: 2.0, IQR 1.0–3.0).

Immune-cell infiltration varied across compartments, with higher densities typically observed at the invasive front compared with the tumor center. CLDN-2 expression was detected in all tumors and distributed between moderate and strong staining intensities. E-cadherin expression varied across tumor compartments, supporting further analysis of spatial adhesion remodeling (Table 1).

### 3.2. CLDN-2 Expression and Clinicopathological Context

**Figure 2:**
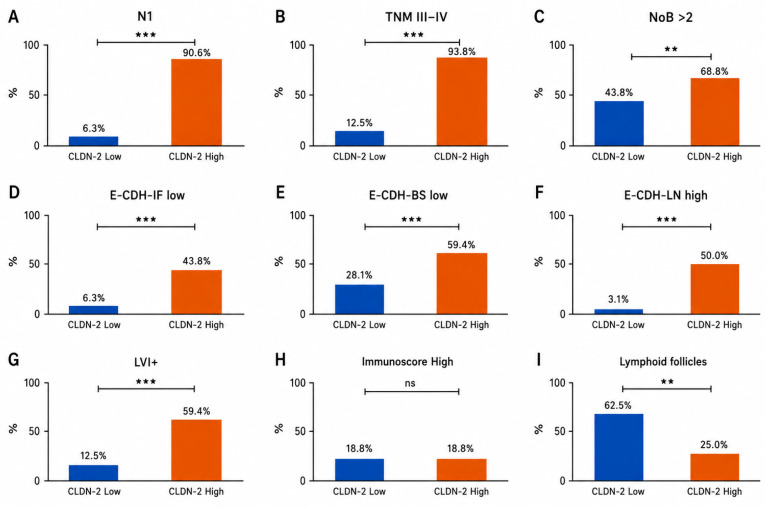
Spatial epithelial remodeling and dissemination-associated histopathological features according to CLDN-2 expression in colorectal cancer. Nine-panel composite illustrating the distribution of selected clinicopathological, invasion-related, epithelial adhesion, vascular invasion, and immune contexture variables according to CLDN-2 expression status (CLDN-2 lower vs. CLDN-2 higher). Bars represent the percentage of cases within each CLDN-2 stratum. (**A**) Lymph node metastases (N+). (**B**) Advanced TNM stage (III–IV). (**C**) Increased tumor budding burden (NoB > 2). (**D**) Reduced membranous E-cadherin expression at the invasive front (E-CDH-IF low). (**E**) Reduced membranous E-cadherin expression in tumor budding sites (E-CDH-BS low). (**F**) Preserved membranous E-cadherin expression in metastatic lymph-node deposits (E-CDH-LN high). (**G**) Presence of lymphovascular invasion (LVI+). (**H**) High Immunoscore distribution. (**I**) Presence of lymphoid follicles. CLDN-2–higher tumors demonstrate consistent enrichment for nodal involvement, advanced stage, tumor budding activity, lymphovascular invasion, and compartment-specific adhesion remodeling, supporting alignment with an invasion-associated epithelial configuration. In contrast, immune-related parameters show weaker differentiation across CLDN-2 expression groups, suggesting partial independence between epithelial structural organization and bulk immune infiltration. Statistical significance between groups is indicated by asterisks (** *p* < 0.01; *** *p* < 0.001; ns, not significant).

Comparison of tumors according to CLDN-2 expression (lower vs. higher) demonstrated that CLDN-2–higher tumors were more frequently associated with nodal involvement and advanced TNM stage (Table 2, Figure 2A–G). In particular, node-positive disease was markedly enriched among CLDN-2–higher tumors, whereas CLDN-2–lower tumors were predominantly node-negative. Differences in distant metastases and primary tumor localization were less pronounced.

In addition, CLDN-2–higher tumors showed a lower prevalence of lymphoid follicles, suggesting that epithelial structural features and immune organization may not be tightly coupled within the same phenotypic axis (Table 2). These observations indicate that CLDN-2 expression aligns preferentially with invasion-related clinicopathological features, while its relationship with immune contexture appears more variable.

### 3.3. CLDN-2 and Invasion-Related Epithelial Remodeling

Tumor budding activity was higher in CLDN-2–higher tumors (Figure 1F) compared with CLDN-2–lower tumors (median 2.0 vs. 1.0 buds, *p* = 0.009), consistent with increased microinvasive potential (Table 2). Complementary histopathological markers of dedifferentiation (TBF, PDC, POR) showed similar directional trends, although these did not reach nominal statistical thresholds.

Compartment-specific analysis of E-cadherin expression revealed a consistent pattern of spatial remodeling associated with CLDN-2 expression (Figure 1E). Representative immunohistochemical patterns of CLDN-2 expression across normal epithelium and invasive tumor compartments are shown in Figure 1A–D. CLDN-2–higher tumors exhibited reduced membranous E-cadherin at the invasive front and within tumor budding areas, indicating localized loss of epithelial cohesion. In contrast, E-cadherin expression in the tumor center remained comparatively preserved, while metastatic lymph-node deposits retained more preserved membranous epithelial organization. This compartment-specific pattern suggests that adhesion remodeling in CRC is spatially regulated rather than uniformly progressive.

**Figure 3:**
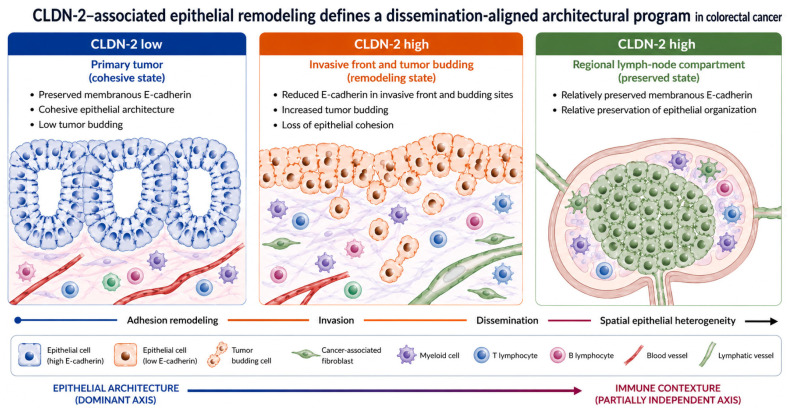
Conceptual representation of spatial epithelial remodeling associated with CLDN-2 expression in colorectal cancer. Schematic, interpretative representation of a spatially organized epithelial remodeling program aligned with CLDN-2 expression. In tumors with lower CLDN-2 expression, membranous E-cadherin is largely preserved, maintaining cohesive epithelial architecture. At the invasive front and within tumor budding regions, higher CLDN-2 expression is associated with reduced membranous E-cadherin expression and localized loss of epithelial cohesion, consistent with increased microinvasive activity and early dissemination-associated remodeling. Within metastatic lymph-node deposits, membranous E-cadherin expression was more frequently retained than within invasion-related tumor regions. This conceptual model integrates spatial adhesion dynamics with invasion-associated epithelial remodeling and summarizes structural relationships observed within the cohort.

These observations are summarized in Figure 3, which provides a conceptual representation of CLDN-2–associated epithelial remodeling across tumor compartments.

Because CLDN-2–higher tumors were strongly enriched for nodal involvement, an exploratory N0-only sensitivity analysis was performed to assess whether CLDN-2–associated epithelial remodeling was already detectable in node-negative disease. Although limited by the small number of CLDN-2–higher N0 tumors, directional consistency was preserved across several invasion-related epithelial features, including higher tumor budding burden and more frequent reduction in membranous E-cadherin at the invasive front and tumor budding sites. These findings should be interpreted cautiously given the limited number of CLDN-2–higher N0 tumors.

### 3.4. Co-Occurrence of Epithelial and Dissemination-Related Features

To examine whether epithelial remodeling variables clustered with dissemination-related histopathological features, we summarized selected binary variables in a co-occurrence matrix (Figure 1G). Higher CLDN-2 expression showed overlap with nodal involvement, increased tumor budding burden, lymphovascular invasion, reduced membranous E-cadherin at the invasive front and budding sites, and preserved membranous E-cadherin expression in regional lymph-node compartments.

This analysis provided a compact representation of how invasion-associated epithelial features co-occurred within the cohort.

### 3.5. Distribution of Compartmental E-Cadherin Scores

Because mean E-cadherin scores may obscure underlying distributional shifts, we further examined the full distribution of compartmental E-cadherin scores according to CLDN-2 status (Figure 1H). CLDN-2–higher tumors showed redistribution toward lower membranous E-cadherin scores at the invasive front and tumor budding sites, consistent with localized loss of epithelial cohesion. In contrast, regional lymph-node compartments more frequently demonstrated preserved membranous E-cadherin expression compared with invasion-related tumor regions.

### 3.6. Pairwise Correlations Among Epithelial and Dissemination-Related Features

Pairwise Spearman correlation analysis was performed to assess relationships among CLDN-2 expression, tumor budding count, lymphovascular invasion, nodal status, and compartment-specific E-cadherin expression (Figure 1I). CLDN-2 expression correlated positively with tumor budding burden, nodal involvement, and preserved membranous E-cadherin expression within metastatic lymph-node deposits, and negatively with membranous E-cadherin expression at the invasive front. Interpretation of correlations involving E-CDH-LN variables requires caution because lymph-node E-cadherin assessment was restricted to tumors with metastatic nodal involvement.

These findings are consistent with a spatially organized pattern in which CLDN-2 expression aligns with invasion-associated epithelial remodeling.

### 3.7. Immune Contexture Patterns

Quantitative immune-cell densities and tumor-associated neutrophil measures did not differ substantially between CLDN-2 strata in either the tumor center or invasive margin. Immunoscore-analog distribution was comparable across groups (Figure 2H). In contrast, lymphoid follicles were less frequent in CLDN-2–higher tumors (Figure 2I). These findings suggest that epithelial structural remodeling and bulk immune infiltration metrics may represent partially distinct dimensions of tumor organization.

## 4. Discussion

### 4.1. CLDN-2 and Invasion-Associated Epithelial Architecture

In the present study, CLDN-2 expression was examined not as an isolated molecular feature but in relation to the structural organization of epithelial architecture in colorectal cancer (CRC). The findings indicate that CLDN-2 expression is associated with a constellation of features consistent with an invasion-associated epithelial configuration, including nodal involvement, increased tumor budding activity, and compartment-specific remodeling of cell–cell adhesion. This perspective is consistent with the concept that CRC progression is not solely driven by molecular alterations but also by coordinated changes in tissue architecture that enable the transition from localized growth to invasion and dissemination [1,2].

Importantly, CLDN-2 does not appear to function as an isolated determinant of tumor behavior but rather aligns with a broader invasion-associated epithelial configuration. Framing CLDN-2 within this architectural context may help reconcile previously heterogeneous observations regarding its role in CRC [9,10,20,34,35,36].

Because CLDN-2–higher tumors were strongly enriched for node-positive and stage III disease, part of the observed architectural alignment may reflect broader progression-associated tumor states rather than CLDN-2-specific biological effects. Nevertheless, exploratory N0-only analyses demonstrated directional preservation of several invasion-related epithelial features, suggesting that CLDN-2-associated architectural remodeling may already be detectable before overt nodal dissemination.

### 4.2. Spatial Remodeling of Adhesion and Tumor Budding

A central observation of this study is the spatially selective remodeling of epithelial adhesion. Reduced membranous E-cadherin expression at the invasive front and within tumor budding areas is consistent with prior studies demonstrating that loss of epithelial cohesion represents a key step in early invasion [7,8,9]. At the same time, membranous E-cadherin expression remained more preserved within metastatic lymph-node deposits than within invasion-related tumor regions, supporting the concept that adhesion remodeling in CRC may be spatially heterogeneous rather than uniformly progressive [13,14,15,37,38]. Importantly, E-cadherin expression within lymph-node compartments was evaluated only in metastatic deposits from node-positive tumors and therefore reflects epithelial characteristics of metastatic CRC tissue rather than uninvolved nodal parenchyma.

The association between CLDN-2 expression and increased tumor budding further reinforces this interpretation. Tumor budding, as a histopathological correlate of early dissemination, reflects localized disruption of epithelial integrity and acquisition of migratory capacity [10,11,12]. In this setting, CLDN-2 expression appears to align with a structural continuum linking localized loss of cohesion at the invasive front to invasion-associated phenotypes, without necessitating complete epithelial–mesenchymal transition [4,5,6] (Figure 3). Tumor budding may also reflect a biological continuum of microinvasive activity associated with nodal dissemination [39,40,41,42]. 

### 4.3. Epithelial Architecture and Immune Contexture as Partially Independent Axes

The results also suggest that epithelial architecture and immune contexture may represent partially independent dimensions of tumor organization. While invasion-related epithelial features—including tumor budding, nodal status, and E-cadherin remodeling—formed a coherent pattern associated with CLDN-2 expression, immune variables such as T-cell densities and lymphoid follicles exhibited greater variability and weaker associations.

This observation is in line with the concept underlying the Immunoscore, which captures an immune-based axis of tumor stratification independent of conventional pathological features [26,27,28]. The lack of strong alignment between epithelial and immune parameters in the present study further supports the notion that these dimensions reflect distinct biological processes that may not be tightly coupled within individual tumors [29,30].

### 4.4. Interpretation of CLDN-2 in the Context of Epithelial Organization

Previous studies have linked CLDN-2 expression to altered barrier function, inflammatory signaling, and proliferative pathways in CRC [9,43]. The present findings do not contradict these observations but rather extend them by placing CLDN-2 within a spatial and structural framework. In this context, CLDN-2 expression may be viewed as part of an epithelial configuration characterized by coordinated adhesion remodeling and increased microinvasive activity.

Emerging claudin-targeted therapeutic strategies further support the translational relevance of claudin family proteins in oncology [44]. The clinical feasibility of claudin-directed therapy has already been demonstrated in gastrointestinal cancers using CLDN18.2-targeted agents [45,46,47].

The conceptual model proposed in Figure 3 should be interpreted as an integrative representation of the spatial relationships observed within the cohort rather than as a defined mechanistic pathway.

### 4.5. Limitations

Several limitations should be acknowledged. First, the study was conducted in a single center and included a relatively small cohort, which limits statistical precision and generalizability. Second, observed associations may partly reflect differences in stage distribution, particularly nodal involvement, and were not formally adjusted for confounding factors. The exploratory N0-only sensitivity analysis was constrained by the small number of CLDN-2–higher node-negative tumors and should therefore be interpreted cautiously. In addition, analyses involving E-cadherin expression within lymph-node compartments were inherently restricted to node-positive tumors and therefore partly conditioned on metastatic status. Third, the absence of survival data precludes assessment of prognostic implications. The cross-sectional design precludes temporal inference regarding whether CLDN-2 upregulation precedes, accompanies, or follows invasion-associated epithelial remodeling. MMR phenotype assessment was based on protein-expression immunohistochemistry without confirmatory molecular MSI testing. Fourth, E-cadherin and CLDN-2 expression were assessed semi-quantitatively by immunohistochemistry, and although evaluation was performed by blinded pathologists with consensus resolution, digital image quantification could strengthen future studies. Finally, the co-occurrence and correlation analyses were exploratory and internally descriptive and were not externally validated.

## 5. Conclusions

In summary, CLDN-2 expression in colorectal cancer is associated with spatial epithelial remodeling characterized by compartment-specific changes in membranous E-cadherin expression and increased tumor budding. Rather than representing an isolated molecular determinant, CLDN-2 expression appears to align with an invasion-associated epithelial configuration characterized by nodal involvement, lymphovascular invasion, and spatial adhesion remodeling.

These findings are hypothesis-generating and warrant validation in independent cohorts.

## Figures and Tables

**Table 1:** Baseline clinicopathological, histopathological, epithelial, and immune characteristics of the study cohort (*n* = 54).

Clinical Variables
Age
<60, *n* = 11 (20.37%) 60–69, *n* = 17 (31.48%) 70–79, *n* = 21 (38.89%) ≥80, *n* = 5 (9.26%)
Gender
Male, *n* = 27 (50%) Female, *n* = 27 (50%)
Primary tumor localization
Rectum, *n* = 23 (42.59%) Sigmoid, *n* = 17 (31.48%) Colon, *n* = 10 (18.52%) Cecum, *n* = 4 (7.41%)
Pathological stage
T stage, depth of invasion
T1, submucosa, *n* = 0 T2, muscularis propria, *n* = 3 (5.55%) T3, subserosa, *n* = 51 (94.45%) T4, serosa or other organs, *n* = 0
N stage, lymph node metastases
N−, absent, *n* = 29 (53.7%) N+, present, *n* = 25 (46.30%)
M stage, distant metastases
M−, absent, *n* = 48 (88.89%) M+, present, *n* = 6 (11.11%)
TNM stage
TNM-I, *n* = 3 (5.55%) TNM-II, *n* = 23 (42.60%) TNM-III, *n* = 22 (40.74%) TNM-IV, *n* = 6 (11.11%)
Histopathological features/invasiveness
Histologic type
Adenocarcinoma, *n* = 53 (98.15%) Mucinous adenocarcinoma, *n* = 1 (1.85%)
Lymphoid follicles (LF)
LF absent, *n* = 30 (55.56%) LF present, *n* = 24 (44.44%)
Lymphovascular invasion (LVI)
LVI-0, absent, *n* = 20 (37.04%) LVI-1, present, *n* = 34 (62.96%)
Perineural invasion (PNI)
PNI-0, absent, *n* = 50 (92.59%) PNI-1, present, *n* = 4 (7.41%)
Tumor budding and dedifferentiation
Tumor budding foci (TBF)
TBF-1, *n* = 50 (92.60%) TBF-2, *n* = 3 (5.55%) TBF-3, *n* = 1 (1.85%)
Poorly differentiated clusters (PDC)
PDC-1, *n* = 34 (62.97%) PDC-2, *n* = 13 (24.07%) PDC-3, *n* = 7 (12.96%)
Areas of poorly differentiated components (POR)
POR-1, *n* = 36 (66.66%) POR-2, *n* = 11 (20.37%) POR-3, *n* = 7 (12.97%)
Number of tumor buds
Median 2.0, IQR 1.0–3.0
Immune microenvironment
Tumor-infiltrating lymphocytes (TIL) CD8+ cells
Tumor center (CD8-TC): median (IQR) = 30.5 (6–97) Invasive front (CD8-IF): median (IQR) = 158 (61–275)
Tumor-infiltrating lymphocytes (TIL) CD3+ cells
Tumor center (CD3-TC): median (IQR) = 9 (1–37) Invasive front (CD3-IF): median (IQR) = 61.5 (16–160)
Tumor-infiltrating lymphocytes (TIL) CD4+ cells
Tumor center (CD4-TC): median (IQR) = 17.5 (2–67) Invasive front (CD4-IF): median (IQR) = 71 (10–159)
Tumor-associated neutrophils (TAN)
Tumor center (TAN-TC): median (IQR) = 24 (7–50) Invasive front (TAN-IF): median (IQR) = 27.5 (8–82)
Immunoscore (IS)
Low, *n* = 26 (48.15%) Intermediate, *n* = 12 (22.22%) High, *n* = 16 (29.63%)
Immunoscore (IS)—binary grouping
Low, *n* = 26 (48.15%) Intermediate + High, *n* = 28 (51.85%)
Epithelial adhesion and barrier markers
Claudin-2 expression pattern (CLDN-2)
CLDN-2-2, moderate, *n* = 25 (46.30%) CLDN-2-3, strong, *n* = 29 (53.7%)
E-cadherin expression pattern in the tumor center (E-CDH-TC)
E-CDH-TC-0, *n* = 1 (1.85%) E-CDH-TC-1, *n* = 8 (14.81%) E-CDH-TC-2, *n* = 16 (29.63%) E-CDH-TC-3, *n* = 29 (53.71%)
E-cadherin expression pattern in the invasive front (E-CDH-IF)
E-CDH-IF-0, *n* = 1 (1.85%) E-CDH-IF-1, *n* = 17 (31.48%) E-CDH-IF-2, *n* = 20 (37.04%) E-CDH-IF-3, *n* = 16 (29.63%)
E-cadherin expression pattern in tumor budding sites (E-CDH-BS)
E-CDH-BS-0, *n* = 12 (22.22%) E-CDH-BS-1, *n* = 18 (33.33%) E-CDH-BS-2, *n* = 8 (14.81%) E-CDH-BS-3, *n* = 16 (29.64%)
E-cadherin expression pattern in lymph-node metastases (E-CDH-LN)
E-CDH-LN-0, *n* = 31 (57.42%) E-CDH-LN-1, *n* = 2 (3.70%) E-CDH-LN-2, *n* = 3 (5.55%) E-CDH-LN-3, *n* = 18 (33.33%)

**Table 2:** Comparison of clinicopathological, invasion-related, epithelial adhesion, and immune variables according to CLDN-2 expression status (CLDN-2 lower vs. CLDN-2 higher). Nominal *p*-values are presented for descriptive comparison. No adjustment for multiple testing was performed.

	Category/Statistic	CLDN-2 Lower (*n* = 25)	CLDN-2 Higher (*n* = 29)	*p*-Value
Clinical variables				
Age (years)	Median (IQR)	70.0 (60.0–75.0)	69.0 (64.0–75.0)	0.828
Sex	Female, *n* (%)	12 (48.0)	15 (51.7)	1.000
	Male, *n* (%)	13 (52.0)	14 (48.3)	
Localization	Rectum, *n* (%)	12 (48.0)	11 (37.9)	0.733
	Sigmoid, *n* (%)	7 (28.0)	10 (34.5)	
	Other colon, *n* (%)	5 (20.0)	5 (17.2)	
	Cecum, *n* (%)	1 (4.0)	3 (10.3)	
Pathological stage				
T stage	T2, *n* (%)	3 (12.0)	0 (0.0)	0.093
	T3, *n* (%)	22 (88.0)	29 (100.0)	
N stage	N−, *n* (%)	24 (96.0)	5 (17.2)	<0.001
	N+, *n* (%)	1 (4.0)	24 (82.8)	
M stage	M−, *n* (%)	23 (92.0)	25 (86.2)	0.675
	M+, *n* (%)	2 (8.0)	4 (13.8)	
TNM stage	I, *n* (%)	3 (12.0)	0 (0.0)	<0.001
	II, *n* (%)	19 (76.0)	4 (13.8)	
	III, *n* (%)	1 (4.0)	21 (72.4)	
	IV, *n* (%)	2 (8.0)	4 (13.8)	
Histopathological features/invasiveness				
PNI	Absent, *n* (%)	23 (92.0)	27 (93.1)	1.000
	Present, *n* (%)	2 (8.0)	2 (6.9)	
Mucinous	Absent, *n* (%)	24 (96.0)	29 (100.0)	0.463
	Present, *n* (%)	1 (4.0)	0 (0.0)	
LVI	Absent, *n* (%)	11 (44.0)	9 (31.0)	0.402
	Present, *n* (%)	14 (56.0)	20 (69.0)	
Lymphoid follicles	Absent, *n* (%)	8 (32.0)	22 (75.9)	0.002
	Present, *n* (%)	17 (68.0)	7 (24.1)	
Tumor budding and dedifferentiation				
Tumor budding foci	1/2/3, *n*	24/1/0	26/2/1	0.570
PDC	1/2/3, *n*	18/6/1	16/7/6	0.175
POR	1/2/3, *n*	20/3/2	16/8/5	0.155
Number of buds (NoB)	median (IQR)	1.0 (1.0–2.0)	2.0 (1.0–3.0)	0.009
Immune infiltration				
CD8-TC	median (IQR)	32.0 (18.0–58.0)	30.0 (15.0–41.0)	0.471
CD8-IF	median (IQR)	133.0 (88.0–219.0)	162.0 (104.0–300.0)	0.252
CD3-TC	median (IQR)	12.0 (1.0–44.0)	6.0 (1.0–25.0)	0.421
CD3-IF	median (IQR)	41.0 (9.0–180.0)	67.0 (20.0–140.0)	0.775
CD4-TC	median (IQR)	20.0 (1.0–67.0)	4.0 (0.0–31.0)	0.071
CD4-IF	median (IQR)	99.0 (34.0–160.0)	67.0 (6.0–170.0)	0.774
TAN-TC	median (IQR)	19.0 (14.0–39.0)	29.0 (9.0–49.0)	0.788
TAN-IF	median (IQR)	15.0 (5.0–60.0)	45.0 (7.0–93.0)	0.549
Immunoscore	Low/Int/High, *n*	13/5/7	13/7/9	0.866
Epithelial adhesion and barrier markers				
E-CDH-BS	0/1/2/3, *n*	9/2/0/14	3/16/8/2	<0.001
E-CDH-TC	0/1/2/3, *n*	1/1/4/19	0/7/12/10	0.007
E-CDH-IF	0/1/2/3, *n*	1/1/11/12	0/16/9/4	<0.001
E-CDH-LN	0/1/2/3, *n*	24/0/0/1	7/2/3/17	<0.001

## Data Availability

The dataset generated and analyzed during the current study is not publicly available due to privacy regulations and consent restrictions but is available from the corresponding author upon reasonable request. All data shared will be de-identified in accordance with ethical guidelines.

## Abbreviations

The following abbreviations are used in this manuscript:

AJCC	American Joint Committee on Cancer
CLDN-2	Claudin-2
CRC	Colorectal Cancer
E-CDH	E-cadherin
E-CDH-BS	E-cadherin at tumor Budding Sites
E-CDH-IF	E-cadherin at the Invasive Front
E-CDH-LN	E-cadherin in Regional Lymph-Nodes
E-CDH-TC	E-cadherin at the Tumor Center
EMT	Epithelial–Mesenchymal Transition
FFPE	Formalin-Fixed, Paraffin-Embedded
H&E	Hematoxylin and Eosin
IF	Invasive Front
IHC	Immunohistochemistry
IQR	Interquartile Range
ITBCC	International Tumor Budding Consensus Conference
LN	Lymph Node
LVI	Lymphovascular Invasion
NoB	Number of Tumor Buds
PDC	Poorly Differentiated Clusters
PNI	Perineural Invasion
POR	Areas of poorly differentiated components
TC	Tumor Center
TBF	Tumor Budding Foci
TIL	Tumor-Infiltrating Lymphocyte
TNM	Tumor–Node–Metastasis
